# Minimally Invasive vs. Open Synovectomy in Rheumatoid Arthritis: Insights into Clinical Recovery, Systemic Inflammation, and Economic Impact

**DOI:** 10.3390/jcm14051519

**Published:** 2025-02-24

**Authors:** Marc-Dan Blajovan, Ahmed Abu-Awwad, Daniel-Laurentiu Pop, Simona-Alina Abu-Awwad, Cristina Tudoran, Daniela Gurgus, Madalina Otilia Timircan, Anca Dinu, Cosmin Ioan Faur

**Affiliations:** 1 Doctoral School, “Victor Babes” University of Medicine and Pharmacy, Eftimie Murgu Square, No. 2, 300041 Timisoara, Romania; marc.blajovan@umft.ro; 2 Department XV—Discipline of Orthopedics—Traumatology, “Victor Babes” University of Medicine and Pharmacy, Eftimie Murgu Square, No. 2, 300041 Timisoara, Romania; daniel.pop@umft.ro (D.-L.P.); or faur17@gmail.com (C.I.F.); 3 “Pius Brinzeu” Emergency Clinical County Hospital, Bld Liviu Rebreanu, No. 156, 300723 Timisoara, Romania; alina.abuawwad@umft.ro (S.-A.A.-A.); tudoran.cristina@umft.ro (C.T.); madalina-otilia.timircan@umft.ro (M.O.T.); 4 Research Center University Professor Doctor Teodor Șora, “Victor Babes” University of Medicine and Pharmacy, Eftimie Murgu Square, No. 2, 300041 Timisoara, Romania; 5 Department XII—Discipline of Obstetrics and Gynecology, “Victor Babes” University of Medicine and Pharmacy, Eftimie Murgu Square, No. 2, 300041 Timisoara, Romania; 6 Department VII, Internal Medicine II, Discipline of Cardiology, “Victor Babes” University of Medicine and Pharmacy, Eftimie Murgu Square, No. 2, 300041 Timisoara, Romania; 7 Center of Molecular Research in Nephrology and Vascular Disease, Faculty of the “Victor Babes” University of Medicine and Pharmacy, Eftimie Murgu Square, No. 2, 300041 Timisoara, Romania; 8 Department of Balneology, Medical Recovery and Rheumatology, Family Discipline, Center for Preventive Medicine, “Victor Babes” University of Medicine and Pharmacy, Eftimie Murgu Square, No. 2, 300041 Timisoara, Romania; gurgus.daniela@umft.ro; 9 Department XVI—Medical Recovery, “Victor Babes” University of Medicine and Pharmacy, Eftimie Murgu Square, No. 2, 300041 Timisoara, Romania; dinu.anca@umft.ro; 10 Research Center for Assessment of Human Motion and Functionality and Disability, “Victor Babes” University of Medicine and Pharmacy, Eftimie Murgu Square, No. 2, 300041 Timisoara, Romania

**Keywords:** rheumatoid arthritis, arthroscopic synovectomy, open synovectomy, systemic inflammation, postoperative recovery, complication rates, cost-effectiveness

## Abstract

**Background:** Rheumatoid arthritis (RA) is a chronic autoimmune disease characterized by persistent synovial inflammation, leading to joint destruction and disability. Synovectomy, the surgical removal of inflamed synovial tissue, is performed when pharmacological treatments are insufficient. This study compares the clinical efficacy, systemic inflammatory response, and cost-effectiveness of minimally invasive arthroscopic synovectomy versus traditional open synovectomy in RA patients. **Methods**: A comparative observational study was conducted on 53 RA patients undergoing either arthroscopic (n = 30) or open synovectomy (n = 23) at “Pius Brînzeu” Timișoara County Emergency Clinical Hospital over nine years. Clinical outcomes, including pain relief (VAS), functional improvement (HAQ), complication rates, and recovery times, were assessed at baseline, 1, 3, 6, and 12 months postoperatively. Systemic inflammatory markers (CRP, IL-6, TNF-α, ESR, and fibrinogen) were measured preoperatively, at 48 h and 30 days postoperatively. A cost-effectiveness analysis evaluated direct and indirect healthcare costs. **Results**: Arthroscopic synovectomy demonstrated significantly faster pain reduction and functional recovery within the first three months (*p* < 0.001), shorter hospital stays (3.1 vs. 6.4 days, *p* < 0.001), and quicker returns to daily activities (14.5 vs. 22.3 days, *p* < 0.001) compared to open synovectomy. Inflammatory markers were significantly lower postoperatively in the arthroscopic group (*p* < 0.01), indicating reduced systemic inflammation. Complication rates were markedly lower in the arthroscopic group (26.66% vs. 82.60%, *p* < 0.001). Despite higher procedural costs, arthroscopic synovectomy proved more cost-effective due to reduced hospitalization and faster recovery. **Conclusions**: Arthroscopic synovectomy offers superior early postoperative outcomes, reduced systemic inflammation, and greater cost-effectiveness compared to open synovectomy, with comparable long-term joint stability. These findings support its preference as the surgical technique of choice for RA patients requiring synovectomy.

## 1. Introduction

Rheumatoid arthritis (RA) is a chronic, systemic autoimmune disease that affects approximately 1% of the global population [1]. Characterized by persistent synovial inflammation, RA leads to pain, joint swelling, and progressive joint destruction, ultimately resulting in significant disability if left untreated [2]. The inflamed synovial membrane thickens and proliferates as the disease progresses, causing irreversible cartilage and bone damage within the affected joints. While pharmacological treatments, including disease-modifying antirheumatic drugs (DMARDs) and biologics, have been instrumental in controlling inflammation and slowing disease progression, some patients continue to experience debilitating symptoms and require surgical intervention to manage joint function and alleviate pain [3].

Synovectomy, the surgical removal of inflamed synovial tissue, has long been employed as a treatment option for RA patients who do not respond adequately to medical therapy. Traditionally, synovectomy has been performed through open surgical techniques, allowing surgeons full access to the joint for extensive synovial tissue removal. However, open synovectomy often involves longer recovery times, increased postoperative pain, and a higher risk of complications such as infection and joint stiffness. In recent decades, advancements in minimally invasive techniques, particularly arthroscopic synovectomy, have provided RA patients with an alternative surgical option [4].

Arthroscopic synovectomy involves smaller incisions, reduces tissue trauma, and is generally associated with faster recovery and fewer postoperative complications, making it an appealing option for many patients and clinicians alike [5].

Despite the potential benefits of minimally invasive techniques, the clinical efficacy of arthroscopic synovectomy compared to traditional open synovectomy in RA patients remains debatable. While some studies suggest that arthroscopic synovectomy offers comparable pain relief and functional improvement with fewer complications [6,7], other research indicates that open synovectomy may be more effective for removing extensive synovial proliferation in advanced cases [8,9,10]. Additionally, while minimally invasive techniques may reduce hospital stays and accelerate recovery, questions remain regarding their long-term outcomes and ability to maintain joint function and stability over time. Given these varying perspectives, a comprehensive comparison of the two techniques is essential to better understand their advantages and limitations.

Although there are studies comparing arthroscopic and open synovectomy in the treatment of rheumatoid arthritis, few have thoroughly analyzed the impact of these techniques on joint stability and long-term quality of life. Additionally, the role of systemic inflammatory markers and the economic impact of these surgical techniques has been largely overlooked, despite their relevance in understanding both physiological recovery and healthcare resource utilization.

The working hypothesis of this study is that arthroscopic synovectomy provides functional outcomes comparable to the open technique but with a lower complication rate, faster recovery, and a reduced systemic inflammatory response due to its minimally invasive nature. Therefore, the primary objective of this research is to compare the efficacy and safety of the two surgical techniques by analyzing key clinical parameters such as complication rates, recovery time, symptom recurrence, and patient satisfaction. Furthermore, this study incorporates the evaluation of inflammatory biomarkers and a cost-effectiveness analysis to provide a comprehensive assessment that extends beyond clinical outcomes. This multidimensional approach aims to optimize clinical decision-making, supporting the selection of the most appropriate surgical technique based on individual patient profiles and healthcare system efficiency.

## 2. Materials and Methods

### 2.1. Study Design and Population

This study was designed as a comparative, observational analysis to evaluate the outcomes of arthroscopic versus open synovectomy in patients with RA. We conducted the study at the “Pius Brînzeu” Timișoara County Emergency Clinical Hospital over 9 years, assessing pain relief, functional improvement, recovery time, complication rates, and long-term joint stability. In total, 53 patients were recruited, 30 underwent arthroscopic synovectomy, and 23 received open synovectomy.

Multiple orthopedic surgical teams performed the surgeries, with team composition varying slightly over the study period. Despite these changes, all surgeons involved had extensive experience in rheumatoid arthritis-related joint surgeries, with a minimum of 5 years of specialized practice and full proficiency in arthroscopic and open synovectomy techniques. The surgical procedures followed standardized protocols to ensure consistency, with all methods strictly adhering to the established principles of synovectomy, including uniform approaches to synovial tissue removal, joint preservation, and hemostasis.

To ensure procedural consistency across different surgeons, all synovectomies—both arthroscopic and open—were performed following a strict institutional protocol, which standardized preoperative planning, the surgical approach, synovial excision techniques, hemostasis methods, and postoperative management. In contrast, all surgeons had a minimum of five years of experience in RA-related joint procedures, regular surgical review meetings were conducted, intraoperative photographic documentation was systematically recorded for quality control, and an independent audit team periodically assessed adherence to these standardized techniques.

Perioperative and postoperative management protocols were standardized across all cases, encompassing preoperative patient optimization, antibiotic prophylaxis, thromboprophylaxis, pain management, and early mobilization strategies. Rehabilitation protocols, including physical therapy regimens and joint function monitoring, were consistently applied throughout the study period, with minor updates reflecting advancements in clinical guidelines [11,12,13,14]. These measures ensured a high degree of consistency in both surgical execution and postoperative care, enhancing the comparability of clinical outcomes across the study population.

Participants were selected based on the following inclusion criteria:

- Patients included in this study presented with moderate to severe rheumatoid arthritis, as defined by the American College of Rheumatology (ACR) criteria [15].
- Persistent synovitis: Active joint inflammation despite optimal pharmacologic therapy (DMARDs or biologics).
- Age range: Adults aged 18 to 75 years.
- Radiographic evidence of joint damage: Presence of erosions or synovial proliferation on MRI or ultrasound.
- Functional limitation: Documented impairment in daily activities due to joint dysfunction.
- Stable medication regimen: No changes in DMARDs or biologics for at least 3 months before surgery.
- Willingness for follow-up: Ability to attend postoperative follow-ups for at least 12 months.
- No prior synovectomy: To avoid bias from previous surgical interventions.
- Good general health: ASA (American Society of Anesthesiologists) physical status classification I–III [16].
- Informed consent: Patients must provide written consent to participate in the study.

Exclusion criteria included:

- History of joint infection: Active or previous septic arthritis in the target joint.
- Severe comorbidities: Including uncontrolled diabetes, advanced cardiovascular disease, or chronic renal failure.
- Concurrent autoimmune diseases, such as lupus, psoriasis, or vasculitis, could confound outcomes.
- Pregnancy or lactation: Due to hormonal effects on joint inflammation and ethical considerations.
- Neurological disorders: Conditions like peripheral neuropathy or stroke that impair joint function independently of RA.
- Malignancy: Active cancer or history of cancer within the past 5 years.
- Coagulopathy: Known bleeding disorders or use of anticoagulant therapy that cannot be safely managed perioperatively.
- Substance abuse: History of drug or alcohol abuse affecting compliance with postoperative care.
- Severe joint deformity: Ankylosis or advanced bone erosion precluding the use of minimally invasive techniques.
- Non-compliance risk: Inability to adhere to postoperative rehabilitation or follow-up protocols.

### 2.2. Data Collection

Data collection for this study was conducted prospectively over 12 months, with information gathered at several key time points: preoperative, postoperative at 1 month, 3 months, 6 months, and at a 1-year follow-up. Data were collected on both demographic characteristics (age, gender, disease duration, body mass index, smoking status, and physical activity level) and clinical variables (duration of DMARD usage, the use of biological therapy, and comorbidities such as hypertension, diabetes, and osteoporosis).

The difference between disease duration and DMARD use duration is attributed to factors such as delayed diagnosis, particularly in cases with atypical onset or slow progression, the initial management with symptomatic therapies (NSAIDs, corticosteroids) before the introduction of DMARDs, as well as interruptions in therapy due to adverse effects, treatment adjustments, or regimen modifications required by disease progression.

Postoperative outcomes assessed included pain levels (using the Visual Analog Scale, VAS), joint function (measured by the Health Assessment Questionnaire, HAQ), recovery duration, hospitalization length, and rates of postoperative complications. Additional long-term outcomes, such as joint stability, recurrence rate, patient satisfaction, and quality of life improvement, were recorded at the one-year mark. Data were systematically entered into a secured electronic database, with appropriate checks to ensure accuracy and completeness.

Inflammatory biomarkers were collected from all participants at three key time points: preoperatively (baseline), at 48 h postoperatively, and at 30 days postoperatively. Blood samples were obtained through standard venipuncture, processed immediately, and stored at controlled temperatures until analysis. The biomarkers assessed included C-reactive protein (CRP), interleukin-6 (IL-6), tumor necrosis factor-alpha (TNF-α), erythrocyte sedimentation rate (ESR), and fibrinogen. The quantification of CRP, IL-6, and TNF-α levels was performed using enzyme-linked immunosorbent assay (ELISA), while ESR and fibrinogen were measured using standard laboratory techniques.

For the cost-effectiveness analysis, both direct and indirect costs were evaluated. Direct costs included surgical expenses, hospitalization costs, postoperative care, and rehabilitation, obtained from hospital billing records. Indirect costs, such as productivity loss due to time off work, were calculated based on patient-reported data and national average income statistics. The total cost per patient was determined by aggregating these components, and comparative analysis was conducted between the two surgical groups to assess economic efficiency.

### 2.3. Surgical Techniques

Arthroscopic synovectomy: This procedure was performed using standard arthroscopic equipment and techniques in the arthroscopic group. Patients received regional anesthesia, and portals were created to access the joint capsule minimally invasively. Arthroscopic instruments were then used to remove inflamed synovial tissue selectively. Care was taken to minimize disruption to surrounding joint structures, allowing for reduced recovery time and fewer complications. The procedure typically took between 1 and 1.5 h, depending on the extent of synovial involvement [17].

Open synovectomy: Patients underwent traditional synovectomy under general anesthesia in the open synovectomy group. A longitudinal incision was made over the affected joint to provide direct access. The complete removal of synovial tissue was performed manually, ensuring thorough synovial excision, which is particularly indicated for advanced or proliferative synovitis cases. The open technique generally took longer, averaging 1.5 to 2 h due to the larger incision and more extensive tissue manipulation required. Following the procedure, sutures were placed, and a standard wound care protocol was implemented [17].

### 2.4. Statistical Analysis

Statistical analysis was performed to rigorously assess the effectiveness of arthroscopic versus open synovectomy in RA patients. Data were analyzed using SPSS version 26.0, with a two-sided *p*-value of <0.05 set as the threshold for statistical significance.

Continuous variables such as age, body mass index (BMI), VAS, and HAQ scores were summarized as mean ± standard deviation. Categorical variables, including gender, smoking status, physical activity levels, and incidence of postoperative complications, were presented as frequencies and percentages. Descriptive statistics allowed for the initial verification of data distribution and enabled the comparison of baseline characteristics between groups.

Independent *t*-tests were used to evaluate differences in continuous variables like age and BMI to ensure comparability between the arthroscopic and open synovectomy groups. To ensure the validity of our comparisons, we assessed the normality of the data using the Shapiro–Wilk test and confirmed the homogeneity of variances with Levene’s test. Chi-square tests assessed categorical variables such as gender distribution and comorbidity prevalence. This step ensured that any postoperative differences observed could be attributed to the surgical technique rather than demographic or clinical disparities.

For continuous outcome variables, including VAS and HAQ scores, repeated measures ANOVA was conducted to examine pain and joint function changes over time at baseline, 1 month, 3 months, 6 months, and 1 year postoperatively. Interaction effects were tested to determine whether the rate of improvement differed significantly between the two groups over time. In cases where sphericity was violated, the Greenhouse–Geisser correction was applied.

Categorical outcome variables, such as complication rates, recurrence rates, and satisfaction scores, were analyzed using Chi-square or Fisher’s exact test, depending on cell sizes, to determine the statistical significance of differences between groups.

To control for potential confounders, multivariate logistic regression models were used to examine the association between surgical technique (arthroscopic vs. open synovectomy) and primary outcomes, adjusting for age, BMI, disease duration, smoking status, and use of biologic therapy. Odds ratios (ORs) with 95% confidence intervals (CIs) were reported to quantify the likelihood of outcomes such as postoperative complications and recurrence in each group. For continuous outcomes, linear regression models were adjusted for these variables to assess further the surgical technique’s impact on postoperative pain reduction and functional improvement.

Any missing data points were evaluated for randomness. If missing data were deemed random, we used multiple imputation techniques to handle the missing values and minimize potential bias. Sensitivity analyses were conducted to ensure the robustness of findings across various imputation scenarios.

### 2.5. Ethical Considerations

This study adhered to the ethical principles of the Declaration of Helsinki and the relevant national and institutional guidelines for human research. Ethical approval was obtained from the Institutional Review Board (IRB) before the study began (106/12 December 2014). All participants provided informed consent after receiving detailed information about the study’s purpose, procedures, risks, and benefits, with the option to withdraw at any time without affecting their medical care.

Participant data were anonymized and securely stored, with access limited to authorized personnel. Data protection followed strict security protocols, ensuring compliance with institutional regulations and GDPR requirements.

Preoperative screenings identified patient-specific risks, and protocols were in place to manage adverse events. Participants were informed about potential surgical risks and had access to the study’s results post-completion. All researchers disclosed any conflicts of interest, and funding sources did not influence the study’s design, data analysis, or publication.

## 3. Results

Our study aimed to compare the outcomes of minimally invasive arthroscopic and traditional open synovectomy in patients with RA across multiple parameters, including pain relief, functional improvement, recovery time, complication rates, and long-term joint stability. A total of 53 patients were recruited, 30 undergoing arthroscopic synovectomy and 23 receiving open synovectomy.

Table 1 provides a detailed breakdown of the study population’s baseline demographic and clinical characteristics in the arthroscopic and open synovectomy groups. These characteristics help establish the comparability of the groups and assess whether demographic or health-related factors could influence postoperative outcomes. Both groups were statistically comparable in terms of age, gender distribution, and disease duration, indicating that any observed differences in outcomes are likely due to the surgical techniques themselves rather than demographic variability. BMI was also similar between the two groups, as were smoking status and physical activity levels, suggesting that lifestyle factors did not vary significantly. Interestingly, although the use of biologic therapies and the duration of DMARD use were relatively consistent across groups, a slightly higher prevalence of comorbidities like hypertension and diabetes was noted in the open synovectomy group. However, these differences were not statistically significant (*p* > 0.05) and are therefore unlikely to introduce significant bias in outcome comparisons. By ensuring that the two groups were balanced in demographics and baseline health status, this study allows for a more focused analysis of the comparative effectiveness of the minimally invasive and traditional open synovectomy techniques in RA treatment.

The evaluation of inflammatory biomarkers revealed distinct differences between the arthroscopic and open synovectomy groups, reflecting variations in the postoperative systemic inflammatory response (Table 2). Key biomarkers, including CRP, IL-6, TNF-α, ESR, and fibrinogen, were assessed at three critical time points: preoperative, 48 h postoperative, and 30 days postoperative.

Both groups exhibited elevated inflammatory markers at 48 h postoperatively, consistent with the acute inflammatory response to surgical trauma. However, significantly higher levels of CRP, IL-6, and TNF-α were observed in the open synovectomy group (*p* < 0.001), indicating a more pronounced inflammatory reaction than the arthroscopic group. This trend persisted at 30 days postoperatively, where inflammatory markers remained elevated in the open synovectomy cohort, although the differences were less pronounced.

Markers of chronic inflammation, such as ESR and fibrinogen, showed a slower decline over time, with values significantly higher in the open synovectomy group at both postoperative time points (*p* < 0.01). These findings suggest that the minimally invasive nature of arthroscopic synovectomy is associated with a reduced inflammatory burden, the faster resolution of systemic inflammation, and potentially lower risks of inflammation-related complications.

Overall, the biomarker profiles support the clinical observation that arthroscopic synovectomy promotes a more favorable postoperative recovery by minimizing the systemic inflammatory response, which may contribute to improved functional outcomes and reduced complication rates.

Postoperative pain was assessed using the VAS, while joint function was evaluated with the HAQ. Both measures were recorded preoperatively and at 1, 3, and 6 months postoperatively (Table 3). Notably, arthroscopic synovectomy patients reported significant pain reduction earlier than those in the open synovectomy group, particularly within the first three months. Regarding functional improvement, patients in the arthroscopic group showed more rapid HAQ improvements, suggesting that minimally invasive procedures might offer faster recovery for joint function.

Recovery duration and hospitalization length were two areas in which arthroscopic synovectomy showed marked advantages (Table 4). Patients in the arthroscopic group had an average hospital stay of 3.1 days compared to 6.4 days in the open synovectomy group. Additionally, patients in the arthroscopic group could resume daily activities significantly sooner than those in the open synovectomy group. The cost-effectiveness analysis compared the direct and indirect costs associated with arthroscopic and open synovectomy, providing insights into the economic impact of both surgical techniques also presented in Table 4. The evaluation included key cost categories such as surgical procedure costs, hospitalization expenses, postoperative care, rehabilitation costs, and productivity loss due to time off work.

While the initial surgical procedure cost was slightly higher for arthroscopic synovectomy (*p* = 0.015), this was offset by significantly lower expenses in subsequent categories. Hospitalization costs were markedly reduced in the arthroscopic group (*p* < 0.001), primarily due to shorter hospital stays and fewer postoperative complications, which also contributed to decreased postoperative care and rehabilitation costs (*p* < 0.001 and *p* = 0.003, respectively).

Furthermore, patients who underwent arthroscopic synovectomy experienced a faster return to work, with significantly fewer days of productivity loss compared to the open synovectomy group (*p* < 0.001). This reduction in indirect costs highlights the broader socioeconomic benefits of minimally invasive surgery beyond the healthcare setting.

When aggregating all cost components, the total cost was significantly lower in the arthroscopic group (*p* < 0.001), despite the higher upfront surgical expenses. These findings demonstrate that arthroscopic synovectomy is not only clinically effective but also cost-efficient, offering economic advantages through reduced hospitalization, quicker recovery, and lower long-term healthcare utilization.

The analysis of postoperative complications revealed a significantly lower overall complication rate in the arthroscopic synovectomy group compared to the open synovectomy group (Table 5). This difference was statistically significant (*p* < 0.001), highlighting the potential safety advantages of the minimally invasive approach. Although individual complications such as infection, joint stiffness, delayed wound healing, postoperative bleeding, and the need for reoperation were observed in both groups, their incidence was consistently lower in the arthroscopic group.

Notably, the infection rate was significantly higher in patients undergoing open synovectomy (*p* = 0.034), suggesting an increased risk likely due to more extensive soft tissue exposure inherent to the open technique. Other complications, including joint stiffness, postoperative bleeding, and delayed wound healing, were more frequent in the open synovectomy group, although the differences did not reach statistical significance. Severe complications such as nerve injury and deep vein thrombosis (DVT) were rare across both groups, with isolated cases recorded only in the open synovectomy cohort. The need for reoperation was infrequent but slightly more common following open procedures.

These findings underscore the favorable safety profile of arthroscopic synovectomy, with reduced postoperative morbidity compared to the open approach. The markedly lower overall complication rate supports the preference for minimally invasive techniques, particularly in patients at higher risk for surgical complications.

The joint stability and symptom recurrence assessment at the 1-year follow-up revealed comparable outcomes between the arthroscopic and open synovectomy groups (Table 6). Although the recurrence rate of symptoms was slightly higher in the arthroscopic group than in the open synovectomy group, this difference was not statistically significant (*p* = 0.686), suggesting that both surgical techniques offer similar long-term efficacy in controlling rheumatoid arthritis-related joint inflammation.

Similarly, joint stability was maintained in most patients across both groups, with a marginally higher stability rate observed following open synovectomy. However, this difference did not reach statistical significance (*p* = 0.484), indicating that arthroscopic synovectomy provides comparable structural outcomes over the long term.

Notably, patient-reported outcomes favored the arthroscopic approach. Patient satisfaction scores were significantly higher in the arthroscopic group (*p* < 0.001), reflecting better subjective experiences likely due to faster recovery, less postoperative discomfort, and lower complication rates. Additionally, the quality of life improvement was notably more excellent in the arthroscopic group (*p* = 0.041), further supporting the positive impact of minimally invasive techniques on overall well-being.

These results suggest that while both procedures achieve similar clinical stability, arthroscopic synovectomy offers superior patient satisfaction and quality of life benefits, making it a favorable option, especially for patients prioritizing postoperative recovery and functional outcomes.

## 4. Discussion

This study compared the clinical outcomes, complication rates, inflammatory responses, and cost-effectiveness of arthroscopic versus open synovectomy in patients with RA. The results indicate that arthroscopic synovectomy provides superior early postoperative outcomes, including faster pain relief, improved functional recovery, lower complication rates, and reduced systemic inflammation, while maintaining comparable long-term joint stability to open synovectomy.

Patients who underwent arthroscopic synovectomy experienced significantly faster pain reduction and functional improvement, particularly within the first three postoperative months. These findings align with previous studies demonstrating that minimally invasive techniques reduce postoperative pain due to less soft tissue trauma and the minimal disruption of periarticular structures [18].

From a pathophysiological perspective, surgical trauma in RA activates the NF-κB signaling pathway, triggering the release of pro-inflammatory cytokines (TNF-α, IL-6, and IL-1β), which exacerbates acute inflammation and contributes to chronic synovial hyperplasia [19,20,21]. Additionally, IL-17, produced by Th17 cells, amplifies synovial inflammation by recruiting neutrophils and is associated with more aggressive disease phenotypes. Matrix metalloproteinases (MMP-1, MMP-3) accelerate joint destruction by degrading extracellular matrix components, while oxidative stress (reactive oxygen species—ROS) stimulates synovial fibroblast proliferation and pannus formation, further driving joint erosion. By minimizing tissue trauma, arthroscopic synovectomy may mitigate these pathological processes and improve postoperative recovery [22,23,24].

The evaluation of systemic inflammatory biomarkers (CRP, IL-6, TNF-α, ESR, and fibrinogen) showed significantly lower levels in the arthroscopic synovectomy group postoperatively, with differences persisting at 30 days. These findings confirm the role of minimally invasive techniques in reducing systemic inflammation [25,26]. The observed differences can be attributed to reduced immune system activation: extensive surgical interventions stimulate the hypothalamic–pituitary–adrenal (HPA) axis and NF-κB pathway, leading to the production of acute-phase reactants (CRP, fibrinogen) and pro-inflammatory cytokines. The less invasive nature of arthroscopy attenuates this inflammatory response, facilitating a faster return to baseline inflammatory levels [27,28].

The observed reduction in systemic inflammation following arthroscopic synovectomy, as evidenced by the lower postoperative levels of CRP, IL-6, and TNF-α, may be attributed not only to reduced surgical trauma but also to distinct immunological pathways. Minimally invasive techniques could mitigate systemic immune activation by decreasing the extent of cytokine release and neuroendocrine stress responses triggered by extensive tissue disruption. Additionally, emerging evidence suggests that different immunomodulatory pathways, including regulatory T-cell activation and localized synovial immune responses, might contribute to the differential inflammatory profile observed between the two surgical techniques. Future studies incorporating immunophenotyping and molecular profiling are warranted to elucidate these mechanisms further [22,25,29].

Beyond inflammatory benefits, arthroscopy was associated with a significantly lower overall complication rate (26.66%) compared to open synovectomy (82.60%), with notable reductions in postoperative infections, bleeding, and delayed wound healing. These findings are consistent with studies showing that smaller incisions and reduced tissue exposure in arthroscopy lower the risk of surgical site infections and wound complications [30,31]. Additionally, DVT was observed exclusively in the open synovectomy group, supporting the Virchow’s triad hypothesis (prolonged immobilization, vascular injury, and hypercoagulability), which is more pronounced in open surgery due to extended recovery times and increased tissue trauma [32,33].

At the one-year follow-up, joint stability and symptom recurrence rates were comparable between the two groups, with no statistically significant differences. However, the open synovectomy group exhibited a slightly lower recurrence rate, aligning with studies suggesting that open techniques allow for more comprehensive synovial tissue excision, reducing the risk of residual disease [34]. Pathophysiologically, residual synovial tissue may serve as a reservoir for chronic inflammatory cells (macrophages, T-cells), sustaining synovial hyperplasia and progressive joint damage. While arthroscopy allows for precise tissue removal, its limited visualization in specific joint compartments may explain the slightly higher recurrence rate observed [35].

From an economic perspective, although arthroscopic synovectomy has higher initial costs, it was found to be more cost-effective in the long term due to shorter hospital stays, faster returns to daily activities, and lower indirect costs, such as reduced productivity loss. These findings are supported by previous studies demonstrating that minimally invasive procedures improve both clinical outcomes and economic efficiency, providing long-term benefits for patients and healthcare systems despite higher upfront costs [36].

This study contributes to the existing literature on synovectomy in RA by integrating clinical outcomes, inflammatory response, and cost-effectiveness analysis. Unlike previous research, we demonstrate that arthroscopic synovectomy accelerates recovery and leads to faster normalization of inflammatory markers. Furthermore, the economic analysis highlights the long-term financial benefits of this technique, suggesting that, despite its higher initial investment, arthroscopy may represent an optimal option both clinically and economically.

From a clinical perspective, our findings support arthroscopic synovectomy as the preferred surgical option for most patients with RA due to its association with faster recovery, lower complication rates, and reduced systemic inflammation, alongside superior cost-effectiveness. However, open synovectomy remains a viable alternative for patients with advanced synovial proliferation or complex joint involvement, as it offers a more comprehensive excision of synovial tissue and may reduce recurrence risk.

A key strength of this study is its integrated approach, combining analyses of clinical outcomes, inflammatory biomarkers, and cost-effectiveness. This methodology provides a deeper understanding of the impact of arthroscopic vs. open synovectomy, going beyond traditional assessments based solely on complication rates and joint function. Additionally, the inclusion of objective inflammatory markers (CRP, IL-6, TNF-α, ESR, and fibrinogen) provides valuable insights into the postoperative immune response, an aspect rarely explored in similar studies. The prospective study design and the standardization of surgical techniques and postoperative protocols contribute to reducing variability and enhancing the applicability of the results in clinical practice.

Despite its strengths, this study has several limitations. First, the relatively short follow-up period (1 year) may not fully capture long-term outcomes, such as joint stability, recurrence rates, and late-onset complications, which are crucial in chronic conditions like rheumatoid arthritis. Second, the sample size is modest, potentially limiting the statistical power to detect subtle differences, especially in rare complications. Additionally, this study did not stratify patients based on disease severity or the presence of comorbidities, which could influence both surgical outcomes and inflammatory responses. The lack of randomization may also introduce selection bias, as patients with more advanced disease may have been preferentially assigned to open synovectomy. Another limitation of this study is the lack of imaging confirmation (MRI or ultrasound) for assessing residual synovitis and the risk of recurrence, which would have provided a more objective measure of persistent inflammation and allowed for a more precise correlation with long-term clinical recurrence. Lastly, the cost-effectiveness analysis was based on data from a single healthcare system, which may limit the generalizability of economic findings to other settings with different healthcare structures and cost models. Future studies should include long-term follow-ups to assess the durability of surgical outcomes, focusing on joint stability, recurrence rates, and late complications beyond one year. Randomized controlled trials are needed to reduce bias and confirm differences in clinical and inflammatory outcomes. Incorporating advanced imaging (MRI, ultrasound) could improve the detection of residual synovial inflammation. This study is limited by a one-year follow-up period, which may not fully capture long-term joint stability and recurrence rates; therefore, future research with extended follow-up beyond 12 months is necessary to assess the durability of surgical outcomes more comprehensively. Additionally, research should explore personalized approaches, identifying patient subgroups that may benefit more from either surgical techniques based on disease severity, comorbidities, or biomarker profiles.

## 5. Conclusions

Arthroscopic synovectomy offers clear advantages over open synovectomy in rheumatoid arthritis management, with faster recovery, reduced pain, and improved joint function in the early postoperative period. It is associated with a lower complication rate (26.66% vs. 82.60%, *p* < 0.001), shorter hospital stays, and an earlier return to daily activities.

The procedure also reduces the systemic inflammatory response, as shown by significantly lower CRP, IL-6, TNF-α, ESR, and fibrinogen levels postoperatively. Despite higher initial costs, arthroscopic synovectomy proves more cost-effective due to fewer complications and quicker recovery.

While both techniques achieve similar long-term joint stability and low recurrence rates, the minimally invasive approach is preferable for most RA patients. Open synovectomy remains valuable for cases with extensive synovial involvement requiring thorough excision.

## Figures and Tables

**Table 1:** Patient demographics and baseline characteristics.

Characteristic	Arthroscopic Synovectomy Group (n = 30)	Open Synovectomy Group (n = 23)	*p*-Value
Mean Age (years)	58.2 ± 9.1	57.6 ± 8.9	0.662
Gender (M/F)	30/50	28/52	0.781
Disease Duration (years)	9.4 ± 3.2	9.6 ± 3.5	0.695
Body Mass Index (BMI) (kg/m^2^)	25.8 ± 4.1	26.1 ± 4.3	0.639
Smoking Status (Current)	6 (20%)	8 (34.78%)	0.346
Physical Activity Level (Low)	23 (76.66%)	18 (78.26%)	0.998
Mean DMARD Use Duration (years)	5.1 ± 1.8	5.4 ± 1.7	0.262
Use of Biologic Therapy (%)	14 (46.66%)	11 (47.82%)	1.000
Hypertension (%)	7 (23.33%)	8 (34.78%)	0.377
Diabetes Mellitus (%)	6 (20%)	4 (17.39%)	0.497
Osteoporosis (%)	5 (16.66%)	4 (17.39%)	1.000

**Table 2:** Inflammatory biomarker assessment.

Biomarker	Evaluation Time Point	Arthroscopic Synovectomy (n = 30)	Open Synovectomy (n = 23)	*p*-Value
CRP (mg/L)	Preoperative	12.5 ± 3.4	13.1 ± 3.9	0.524
	48 h Postoperative	35.7 ± 8.2	52.4 ± 10.6	<0.001
	30 Days Postoperative	7.8 ± 2.1	11.4 ± 3.3	0.004
IL-6 (pg/mL)	Preoperative	18.9 ± 5.6	19.7 ± 6.2	0.611
	48 h Postoperative	42.3 ± 9.4	68.2 ± 12.8	<0.001
	30 Days Postoperative	10.2 ± 3.1	15.6 ± 4.5	0.002
TNF-α (pg/mL)	Preoperative	14.5 ± 4.1	15.2 ± 4.8	0.483
	48 h Postoperative	28.6 ± 6.9	44.3 ± 9.5	<0.001
	30 Days Postoperative	9.4 ± 2.7	13.8 ± 3.9	0.003
ESR (mm/h)	Preoperative	28 ± 6	30 ± 7	0.327
	48 h Postoperative	42 ± 9	58 ± 11	<0.001
	30 Days Postoperative	18 ± 5	25 ± 6	0.002
Fibrinogen (mg/dL)	Preoperative	350 ± 50	360 ± 55	0.452
	48 h Postoperative	480 ± 70	620 ± 85	<0.001
	30 Days Postoperative	310 ± 40	400 ± 60	0.001

**Table 3:** Pain relief and functional improvement.

Time Point	Arthroscopic Synovectomy (N = 30)	Open Synovectomy (N = 23)	*p*-Value
VAS			
Preoperative	7.8 ± 1.1	7.9 ± 1.0	0.533
1 month	4.2 ± 1.2	5.7 ± 1.4	<0.001
3 months	3.6 ± 1.0	4.8 ± 1.2	<0.01
6 months	3.2 ± 1.1	3.4 ± 1.3	0.275
HAQ			
Preoperative	2.4 ± 0.6	2.5 ± 0.5	0.537
1 month	1.8 ± 0.5	2.3 ± 0.6	<0.001
3 months	1.5 ± 0.4	1.9 ± 0.5	<0.01
6 months	1.4 ± 0.4	1.6 ± 0.5	0.004

**Table 4:** Recovery time and hospitalization and cost-effectiveness analysis.

Outcome	Arthroscopic Synovectomy (n = 30)	Open Synovectomy (n = 23)	*p*-Value
Average Hospital Stay (days)	3.1 ± 1.2	6.4 ± 1.8	<0.001
Time to Resume Daily Activities (days)	14.5 ± 3.1	22.3 ± 4.2	<0.001
Cost category			
Surgical Procedure Cost (EUR)	3.211 ± 452	2.845 ± 399	0.015
Hospitalization Cost (EUR)	1.521 ± 310	2.700 ± 499	<0.001
Postoperative Care Cost (EUR)	907 ± 151	1.409 ± 251	<0.001
Rehabilitation Cost (EUR)	603 ± 120	903 ± 180	0.003
Productivity Loss (Days Off Work)	12 ± 4	21 ± 6	<0.001
Total Cost (EUR)	6.230 ± 859	7.810 ± 1.245	<0.001

**Table 5:** Complication rates and types.

Complication	Arthroscopic Synovectomy (n = 30)	Open Synovectomy (n = 23)	*p*-Value
Infection	1 (3.33%)	6 (26.08%)	0.034
Joint Stiffness	2 (6.66%)	4 (17.39%)	0.384
Delayed Wound Healing	1 (3.33%)	2 (8.69%)	0.572
Postoperative Bleeding	2 (6.66%)	3 (13.04%)	0.642
Nerve Injury	1 (3.33%)	1 (4.34%)	1.00
DVT	0 (0%)	1 (4.34%)	0.434
Reoperation Required	1 (3.33%)	2 (8.69%)	0.572
Overall Complication Rate	8 (26.66%)	19 (82.60%)	<0.001

**Table 6:** Joint stability and symptom recurrence were assessed at a 1-year follow-up.

Outcome	Arthroscopic Synovectomy (N = 30)	Open Synovectomy (N = 23)	*p*-Value
Symptom recurrence (1-year follow-up)	4 (13.33%)	2 (8.69%)	0.686
Joint stability (1-year follow-up)	23 (76.66%)	20 (86.95%)	0.484
Patient satisfaction (scale of 1–10)	8.7 ± 1.1	7.4 ± 1.3	<0.001
Quality of life improvement (%)	24 (80.00%)	12 (52.17%)	0.041

## Data Availability

The datasets used and analyzed during the current study are available from the first author.
